# Supplementary material for: Rare and declining bird species benefit most from designating protected areas for conservation in the UK
Source: Nat Ecol Evol. 2022 Dec 5;7(1):92–101. doi: 10.1038/s41559-022-01927-4 (PMC9834046; doi:10.1038/s41559-022-01927-4)
Supplement: Supplementary file 1 — Supplementary Tables S1-S8, Supplementary Figures S1-S2 [file 41559_2022_1927_MOESM1_ESM.pdf]

nature ecology & evolution

Article https://doi.org/10.1038/s41559-022-01927-4

Rare and declining bird species benefit most from designating protected areas for conservation in the UK

In the format provided by the authors and unedited

Supplementary Table 1 Percentage of bird species influenced by extent of protected area, dividing species into those with significant and non-significant (in parentheses) positive and negative correlations between population measures and area of designation. The χ^2 and P values relate to a two-sided test of proportion, testing the difference in proportion of species with significantly positive or negative correlations with area of designation. Significant results ($P < 0.05$) are shown in bold.

	**All Protected Areas**					**SSSI**					**SPA**					**SAC**				
	**+**	**(+)**	**(-)**	**-**	**χ^2, P**	**+**	**(+)**	**(-)**	**-**	**χ^2, P**	**+**	**(+)**	**(-)**	**-**	**χ^2, P**	**+**	**(+)**	**(-)**	**-**	**χ^2, P**
**Occurrence** (n=179)	**47.7**	**16.9**	**14.5**	**21.0**	**17.16, <0.001**	**48.8**	**17.8**	**11.3**	**22.0**	**16.27, <0.001**	40.9	13.0	16.6	29.6	2.72, 0.099	27.6	21.8	24.7	25.9	0.04, 0.834
**Colonisation** (n=165)	24.7	26.5	22.3	26.5	0.05, 0.830	25.2	28.8	20.6	25.3	>0.01, 1	22.4	25.4	26.0	26.0	0.3, 0.581	**11.1**	**30.4**	**31.0**	**27.5**	**11.05, 0.001**
**Persistence** (n=130)	30.4	31.3	18.0	20.3	2.22, 0.137	28.9	32.0	20.3	18.8	2.36, 0.124	18.6	41.1	23.2	17.1	0.02, 0.883	20.0	34.6	30.0	15.4	0.54, 0.461
**Abundance** (n=133)	**48.1**	**18.0**	**8.3**	**25.6**	**8.58, 0.003**	**48.1**	**19.6**	**7.5**	**24.8**	**9.28, 0.002**	39.1	21.8	14.3	24.8	3.81, 0.051	36.8	18.0	21.0	24.1	3.16, 0.075
**Abundance Trend** (n=133)	20.3	27.8	29.3	22.5	0.07, 0.791	20.3	27.8	29.3	22.6	0.07, 0.791	21.8	36.8	29.3	12.0	3.2, 0.074	21.0	21.8	30.8	26.3	0.57, 0.450
**Productivity** (n=22)	**9.9**	**22.7**	**22.7**	**45.5**	**4.08, 0.043**	**9.9**	**22.7**	**22.7**	**45.5**	**4.08, 0.043**	13.6	45.5	18.1	22.7	1.26, 0.724	9.8	27.2	22.7	40.9	2.27, 0.070
**Productivity Trend** (n=22)	13.6	31.8	22.7	31.8	0.9, 0.343	13.6	27.3	27.3	31.8	0.9, 0.34	13.6	27.3	22.7	36.4	1.45, 0.228	18.1	31.8	18.2	31.8	0.36, 0.547

Supplementary Table 2 Mean association between demographic characteristics of individual species and protected area extent within the survey square (± standard error), and (final column) the results of the two-sided t-test comparing the mean of SPA with SAC. P values are shown after each statistic and significant results (P < 0.05) are shown in bold.

	N Spp	All PA	SSSI	SPA	SAC	SPA vs SAC
**Occurrence**	179	**0.49 ± 0.07, >0.001**	**0.48 ± 0.07, >0.001**	**0.31 ± 0.07, >0.001**	**0.13 ± 0.06, 0.032**	**t = 3.5, >0.001**
**Colonisations**	165	**0.27 ± 0.08, 0.001**	**0.29 ± 0.08, 0.001**	0.15 ± 0.08, 0.073	-0.05 ± 0.08, 0.566	**t = 3.6, >0.001**
**Persistence**	130	**0.23 ± 0.09, 0.012**	**0.26 ± 0.1, 0.008**	0.15 ± 0.11, 0.183	0.12 ± 0.09, 0.198	t = 1.7, 0.085
**Abundance**	133	**0.25 ± 0.05, >0.001**	**0.26 ± 0.05, >0.001**	**0.19 ± 0.05, 0.001**	**0.12 ± 0.05, 0.023**	t = 1.7, 0.098
**Abundance trend**	133	0.01 ± 0.04, 0.76	0.02 ± 0.04, 0.722	0.09 ± 0.05, 0.075	-0.03 ± 0.06, 0.682	**t = 2.3, 0.022**
**Productivity**	22	**-0.18 ± 0.07, 0.022**	**-0.18 ± 0.07, 0.018**	-0.11 ± 0.08, 0.211	-0.25 ± 0.12, 0.054	t = -1.0, 0.319
**Productivity trend**	22	-0.07 ± 0.07, 0.294	-0.07 ± 0.07, 0.308	-0.06 ± 0.1, 0.549	0.21 ± 0.19, 0.269	t = 1.5, 0.142

Supplementary Table 3. Mean association between the relationship of demographic parameters to PA extent and that of productivity to PA extent for 22 species. The final row compares the association of PA and abundance trend with that of trend in productivity and PA extent. Slope coefficient parameters ± standard errors from a GLM are shown, and two-sided P values are shown after each value. Significant results (P < 0.05) are shown in bold.

Parameter	All PA	SSSI	SPA	SAC
**Occupancy**	0.59 ± 0.44, 0.188	0.59 ± 0.47, 0.222	0.57 ± 0.30, 0.07	-0.21 ± 0.18, 0.267
**Colonisations**	0.61 ± 0.47, 0.206	0.57 ± 0.51, 0.264	0.57 ± 0.30, 0.075	-0.286 ± 0.2, 0.168
**Persistence**	0.73 ± 0.45, 0.123	0.75 ± 0.47, 0.131	0.46 ± 0.29, 0.129	-0.327 ± 0.26, 0.227
**Abundance**	0.55 ± 0.42, 0.207	0.57 ± 0.43, 0.203	**0.61 ± 0.26, 0.03**	**-0.40 ± 0.18, 0.038**
**Abundance Trend**	-0.38 ± 0.19, 0.056	-0.39 ± 0.19, 0.054	-0.43 ± 0.29, 0.065	0.12 ± 0.15, 0.429
**Abundance Trend (vs Trend)**	0.34 ± 0.21, 0.12	0.36 ± 0.21, 0.101	**0.40 ± 0.18, 0.038**	0.05 ± 0.10, 0.624

Supplementary Table 4 Model estimates of the relationship between extent of designated protected area and species population metrics. Estimates are quoted as mean and 95% CI's, significant relationships are in bold.

	All PA	SSSI	SPA	SAC
**Occurrence**				
Log Mass	-0.213 (-0.527, 0.11)	-0.217 (-0.499, 0.091)	-0.081 (-0.444, 0.237)	**-0.326 (-0.534, -0.097)**
Log Pop Size	**-1.069 (-1.322, -0.804)**	**-1.009 (-1.274, -0.707)**	**-0.946 (-1.233, -0.702)**	**-0.726 (-0.978, -0.498)**
Log Pop Change	**-0.23 (-0.442, -0.027)**	**-0.252 (-0.465, -0.029)**	**-0.223 (-0.425, -0.022)**	-0.093 (-0.276, 0.116)
SSI	**0.487 (0.252, 0.771)**	**0.480 (0.185, 0.730)**	**0.420 (0.159, 0.660)**	0.253 (-0.006, 0.455)
STI	**0.368 (0.094, 0.627)**	**0.366 (0.105, 0.670)**	0.249 (-0.024, 0.509)	-0.062 (-0.283, 0.181)
Wetland	**0.575 (0.083, 1.021)**	**0.585 (0.161, 0.989)**	0.352 (-0.214, 0.856)	0.178 (-0.111, 0.464)
Upland	0.235 (-0.181, 0.686)	0.291 (-0.009, 0.668)	0.155 (-0.368, 0.657)	0.133 (-0.095, 0.358)
Coastal	0.457 (-0.074, 0.945)	**0.522 (0.090, 0.918)**	0.321 (-0.282, 0.857)	-0.065 (-0.382, 0.237)
Farmland	0.387 (-0.058, 0.803)	**0.363 (0, 0.751)**	0.285 (-0.256, 0.807)	0.148 (-0.096, 0.392)
Woodland	**0.508 (0.076, 0.957)**	**0.449 (0.100, 0.810)**	0.214 (-0.300, 0.729)	0.216 (-0.003, 0.435)
Urban	-0.222 (-0.859, 0.375)	-0.296 (-0.900, 0.304)	-0.200 (-0.831, 0.491)	-0.347 (-0.810, 0.127)
Unclassified	**0.579 (0.100, 1.032)**	**0.601 (0.192, 0.978)**	0.297 (-0.252, 0.796)	0.238 (-0.020, 0.509)
**Colonisations**				
Log Mass	-0.215 (-0.532, 0.050)	-0.214 (-0.461, 0.048)	-0.084 (-0.418, 0.213)	-0.276 (-0.463, -0.097)
Log Pop Size	**-1.143 (-1.410, -0.914)**	**-1.121 (-1.378, -0.864)**	**-0.990 (-1.250, -0.743)**	**-0.835 (-1.051, -0.645)**
Log Pop Change	-0.187 (-0.403, 0.015)	**-0.235 (-0.460, -0.036)**	**-0.215 (-0.436, -0.015)**	-0.100 (-0.275, 0.09)
SSI	**0.477 (0.238, 0.748)**	**0.529 (0.268, 0.758)**	**0.517 (0.258, 0.756)**	**0.344 (0.150, 0.557)**
STI	**0.280 (0.037, 0.563)**	0.237 (-0.023, 0.491)	0.175 (-0.090, 0.428)	-0.141 (-0.354, 0.060)
Wetland	0.274 (-0.151, 0.648)	0.317 (-0.012, 0.644)	0.121 (-0.351, 0.561)	-0.005 (-0.229, 0.233)
Upland	0.064 (-0.286, 0.423)	0.046 (-0.218, 0.334)	-0.010 (-0.445, 0.417)	-0.100 (-0.266, 0.075)
Coastal	0.262 (-0.159, 0.646)	**0.389 (0.011, 0.737)**	0.213 (-0.292, 0.671)	-0.260 (-0.529, 0.001)
Farmland	0.301 (-0.041, 0.688)	**0.334 (0.025, 0.671)**	0.301 (-0.127, 0.731)	**0.188 (0.003, 0.392)**
Woodland	0.301 (-0.053, 0.682)	0.261 (-0.003, 0.582)	0.151 (-0.258, 0.564)	0.125 (-0.051, 0.313)
Urban	-0.319 (-0.850, 0.201)	-0.338 (-0.816, 0.147)	-0.218 (-0.744, 0.343)	-0.316 (-0.64, 0.005)
Unclassified	0.326 (-0.069, 0.688)	**0.375 (0.066, 0.672)**	0.187 (-0.247, 0.584)	0.097 (-0.073, 0.284)

Persistence

Log Mass	-0.328 (-0.64, 0.009)	**-0.359 (-0.688, -0.043)**	-0.251 (-0.569, 0.049)	**-0.406 (-0.702, -0.108)**
Log Pop Size	**-0.576 (-0.863, -0.294)**	**-0.565 (-0.844, -0.282)**	**-0.375 (-0.662, -0.107)**	**-0.441 (-0.688, -0.191)**
Log Pop Change	**-0.303 (-0.568, -0.064)**	**-0.339 (-0.591, -0.072)**	**-0.245 (-0.523, -0.004)**	**-0.440 (-0.696, -0.200)**
SSI	**0.448 (0.122, 0.785)**	**0.486 (0.135, 0.812)**	**0.565 (0.249, 0.862)**	**0.369 (0.061, 0.696)**
STI	-0.009 (-0.348, 0.303)	0.059 (-0.297, 0.393)	0.011 (-0.326, 0.326)	-0.141 (-0.456, 0.183)
Wetland	**0.671 (0.239, 1.119)**	**0.678 (0.241, 1.116)**	**0.583 (0.137, 1.007)**	**0.48 (0.071, 0.898)**
Upland	0.166 (-0.158, 0.535)	0.242 (-0.073, 0.589)	0.093 (-0.249, 0.441)	0.252 (-0.021, 0.548)
Coastal	0.136 (-0.325, 0.596)	0.23 (-0.215, 0.666)	0.122 (-0.329, 0.559)	0.014 (-0.412, 0.46)
Farmland	0.240 (-0.073, 0.602)	0.198 (-0.124, 0.563)	0.302 (-0.025, 0.659)	0.250 (-0.036, 0.563)
Woodland	**0.384 (0.069, 0.719)**	**0.354 (0.060, 0.677)**	0.280 (-0.032, 0.596)	0.229 (-0.05, 0.519)
Urban	-0.42 (-0.927, 0.116)	-0.458 (-0.952, 0.069)	-0.445 (-0.947, 0.038)	-0.336 (-0.774, 0.119)
Unclassified	0.319 (-0.018, 0.676)	**0.345 (0.015, 0.687)**	**0.380 (0.032, 0.728)**	**0.378 (0.084, 0.684)**

Abundance

Log Mass	-0.100 (-0.325, 0.108)	-0.094 (-0.308, 0.138)	-0.096 (-0.326, 0.140)	-0.136 (-0.350, 0.093)
Log Pop Size	**-0.401 (-0.617, -0.188)**	**-0.387 (-0.595, -0.142)**	**-0.388 (-0.614, -0.151)**	**-0.411 (-0.626, -0.194)**
Log Pop Change	-0.053 (-0.222, 0.149)	-0.048 (-0.249, 0.131)	0.004 (-0.108, 0.205)	0.032 (-0.140, 0.232)
SSI	**0.412 (0.203, 0.642)**	**0.427 (0.188, 0.644)**	**0.466 (0.231, 0.693)**	**0.374 (0.164, 0.613)**
STI	0.051 (-0.154, 0.291)	0.0440 (-0.200, 0.260)	-0.113 (-0.353, 0.131)	0.034 (-0.175, 0.256)
Wetland	**0.494 (0.174, 0.799)**	**0.493 (0.188, 0.787)**	**0.339 (0.030, 0.647)**	0.125 (-0.174, 0.412)
Upland	0.236 (-0.003, 0.482)	**0.266 (0.032, 0.531)**	0.142 (-0.108, 0.414)	0.214 (-0.018, 0.446)
Coastal	**0.500 (0.128, 0.864)**	**0.485 (0.096, 0.870)**	0.263 (-0.132, 0.663)	**0.483 (0.112, 0.846)**
Farmland	-0.017 (-0.265, 0.229)	-0.008 (-0.266, 0.256)	0.111 (-0.135, 0.366)	-0.004 (-0.236, 0.235)
Woodland	**0.336 (0.126, 0.564)**	**0.351 (0.133, 0.594)**	**0.212 (0.001, 0.444)**	0.161 (-0.039, 0.365)
Urban	**-0.684 (-1.088, -0.242)**	**-0.683 (-1.127, -0.241)**	-0.303 (-0.747, 0.109)	**-0.802 (-1.21, -0.415)**
Unclassified	**0.429 (0.181, 0.691)**	**0.446 (0.182, 0.718)**	**0.394 (0.136, 0.677)**	**0.398 (0.163, 0.649)**

Trend

Log Mass	**-0.215 (-0.402, -0.040)**	**-0.212 (-0.396, -0.033)**	-0.153 (-0.367, 0.079)	**-0.468 (-0.735, -0.169)**
Log Pop Size	-0.037 (-0.224, 0.145)	-0.025 (-0.214, 0.159)	-0.024 (-0.236, 0.175)	-0.107 (-0.353, 0.166)
Log Pop Change	**-0.282 (-0.430, -0.126)**	**-0.290 (-0.45, -0.132)**	**-0.332 (-0.504, -0.142)**	-0.215 (-0.44, 0.017)
SSI	-0.022 (-0.213, 0.152)	-0.012 (-0.206, 0.167)	0.022 (-0.188, 0.237)	-0.069 (-0.304, 0.201)
STI	-0.169 (-0.353, 0.023)	-0.158 (-0.369, 0.022)	-0.022 (-0.242, 0.194)	**-0.382 (-0.650, -0.107)**
Wetland	0.216 (-0.031, 0.476)	0.201 (-0.044, 0.477)	**0.329 (0.019, 0.640)**	0.280 (-0.099, 0.686)

Upland	-0.071 (-0.261, 0.125)	-0.061 (-0.268, 0.151)	0.053 (-0.166, 0.290)	-0.15 (-0.459, 0.189)
Coastal	-0.043 (-0.344, 0.263)	-0.017 (-0.344, 0.300)	0.110 (-0.254, 0.489)	-0.237 (-0.674, 0.27)
Farmland	0.038 (-0.167, 0.232)	0.033 (-0.178, 0.238)	-0.005 (-0.238, 0.257)	0.062 (-0.234, 0.354)
Woodland	-0.141 (-0.311, 0.043)	-0.140 (-0.318, 0.058)	-0.010 (-0.231, 0.222)	-0.202 (-0.479, 0.085)
Urban	**0.328 (0.005, 0.659)**	0.309 (-0.037, 0.667)	**0.414 (0.023, 0.853)**	0.747 (0.25, 1.248)
Unclassified	**0.209 (0.006, 0.413)**	0.204 (0.002, 0.438)	0.221 (-0.027, 0.464)	0.11 (-0.209, 0.415)

Supplementary Table 5 Mean effect of extent of protected areas on population metrics of species of conservation concern (BoCC) or which are legally protected (Annex/Schedule 1). Asterisks indicate the significance of the difference in response to PA between species of conservation concern (red/amber-listed) and those not (green-listed) or for those designated under Annex 1 ('Birds' Directive) and Schedule 1 (UK Wildlife & Countryside Act) and those not so designated (exact P values are after the brackets, and significant results, P < 0.05, are shown in bold.

	All PA	SSSI	SPA	SAC
**Occurrence**				
BoCC Green	-0.08 (-0.18, 0.02)	-0.07 (-0.18, 0.04)	-0.242 (-0.35, -0.14)	-0.141 (-0.23, -0.05)
BoCC Amber	**0.235 (0.08, 0.39), 0.001**	**0.273 (0.11, 0.44), 0.001**	**0.196 (0.04, 0.35), < 0.001**	**0.08 (-0.05, 0.21), 0.008**
BoCC Red	0.077 (-0.17, 0.32), 0.249	0.147 (-0.12, 0.41), 0.142	-0.026 (-0.28, 0.22), 0.122	0.005 (-0.22, 0.23), 0.23
Not Annex 1	-0.004 (-0.09, 0.08)	0.017 (-0.07, 0.11)	-0.135 (-0.22, -0.05)	-0.091 (-0.16, -0.02)
Annex 1	**0.563 (0.2, 0.93), 0.003**	**0.652 (0.25, 1.05), 0.003**	**0.605 (0.26, 0.95), < 0.001**	**0.41 (0.11, 0.71), 0.002**
Not Schedule 1	0.001 (-0.08, 0.08)	0.024 (-0.07, 0.11)	-0.122 (-0.21, -0.04)	-0.082 (-0.15, -0.01)
Schedule 1	**0.639 (0.21, 1.07), 0.004**	**0.688 (0.22, 1.16), 0.007**	**0.58 (0.16, 1), 0.001**	**0.399 (0.03, 0.77), 0.013**
**Colonisation**				
BoCC Green	-0.336 (-0.44, -0.23)	-0.342 (-0.45, -0.23)	-0.397 (-0.51, -0.29)	-0.366 (-0.45, -0.28)
BoCC Amber	**0.054 (-0.11, 0.21), < 0.001**	**0.086 (-0.08, 0.25), < 0.001**	**0.087 (-0.07, 0.25), < 0.001**	**-0.098 (-0.23, 0.03), 0.001**
BoCC Red	-0.088 (-0.36, 0.18), 0.1	**-0.026 (-0.31, 0.26), 0.044**	-0.103 (-0.38, 0.17), 0.055	-0.164 (-0.39, 0.06), 0.099

Not Annex 1	-0.238 (-0.32, -0.15)	-0.229 (-0.32, -0.14)	-0.272 (-0.36, -0.18)	-0.297 (-0.37, -0.23)
Annex 1	**0.622 (0.21, 1.03), < 0.001**	**0.677 (0.25, 1.11), < 0.001**	**0.655 (0.26, 1.05), < 0.001**	**0.29 (-0.03, 0.61), 0.001**
Not Schedule 1	-0.236 (-0.32, -0.15)	-0.226 (-0.32, -0.14)	-0.263 (-0.35, -0.17)	-0.294 (-0.36, -0.22)
Schedule 1	**0.672 (0.24, 1.11), < 0.001**	**0.727 (0.27, 1.18), < 0.001**	**0.594 (0.17, 1.02), < 0.001**	**0.319 (-0.03, 0.67), 0.001**
**Persistence**				
BoCC Green	-0.066 (-0.2, 0.07)	-0.047 (-0.19, 0.1)	-0.085 (-0.22, 0.05)	-0.066 (-0.19, 0.06)
BoCC Amber	0.034 (-0.18, 0.25), 0.445	0.062 (-0.16, 0.29), 0.423	**0.221 (0.02, 0.42), 0.013**	0.125 (-0.07, 0.32), 0.103
BoCC Red	0.049 (-0.29, 0.39), 0.533	0.091 (-0.26, 0.44), 0.474	-0.041 (-0.36, 0.27), 0.803	0.155 (-0.15, 0.46), 0.193
Not Annex 1	-0.041 (-0.15, 0.07)	-0.016 (-0.13, 0.1)	-0.013 (-0.12, 0.09)	-0.009 (-0.11, 0.09)
Annex 1	0.625 (-0.15, 1.4), 0.1	0.628 (-0.2, 1.45), 0.132	**0.709 (0.04, 1.37), 0.037**	**0.69 (0.07, 1.32), 0.032**
Not Schedule 1	-0.031 (-0.14, 0.08)	-0.007 (-0.12, 0.11)	0.003 (-0.1, 0.11)	0.006 (-0.09, 0.11)
Schedule 1	0.543 (-0.88, 1.97), 0.431	0.538 (-0.97, 2.05), 0.481	0.26 (-1.04, 1.56), 0.701	0.343 (-0.9, 1.59), 0.596
**Abundance**				
BoCC Green	-0.13 (-0.23, -0.04)	-0.13 (-0.23, -0.03)	-0.21 (-0.31, -0.12)	-0.18 (-0.27, -0.09)
BoCC Amber	-0.22 (-0.39, -0.05), 0.391	-0.22 (-0.40, -0.04), 0.393	-0.14 (-0.31, 0.02), 0.481	-0.18 (-0.33, -0.03), 0.994

BoCC Red	-0.04 (-0.32, 0.23), 0.539	-0.05 (-0.33, 0.23), 0.599	-0.03 (-0.29, 0.23), 0.210	-0.04 (-0.28, 0.21), 0.276
Not Annex 1	-0.15 (-0.23, -0.07)	-0.15 (-0.23, -0.07)	-0.19 (-0.27, -0.11)	-0.18 (-0.25, -0.11)
Annex 1	0.56 (-0.18, 1.30), 0.063.	0.59 (-0.18, 1.35), 0.061	**0.57 (-0.04, 1.17), 0.017**	**0.61 (0.04, 1.17), 0.008**
Not Schedule 1	-0.15 (-0.23, -0.07)	-0.15 (-0.23, -0.07)	-0.19 (-0.27, -0.11)	-0.18 (-0.25, -0.10)
Schedule 1	0.47 (-0.35, 1.29), 0.139	0.48 (-0.36, 1.32), 0.147	0.47 (-0.21, 1.16), 0.061	**0.54 (-0.11, 1.19), 0.035**
**Trend**				
BoCC Green	-0.03 (-0.09, 0.02)	-0.03 (-0.09, 0.02)	0.05 (-0.01, 0.11)	-0.08 (-0.15, -0.00)
BoCC Amber	0.01 (-0.09, 0.10), 0.474	0.01 (-0.08, 0.10), 0.445	0.07 (-0.03, 0.17), 0.676	-0.03 (-0.16, 0.09), 0.536
BoCC Red	0.10 (-0.04, 0.25), 0.083	0.09 (-0.05, 0.24), 0.114	0.10 (-0.06, 0.26), 0.533	0.11 (-0.09, 0.31), 0.084
Not Annex 1	-0.01 (-0.05, 0.03)	-0.01 (-0.06, 0.03)	0.06 (0.01, 0.11)	-0.05 (-0.11, 0.02)
Annex 1	-0.13 (-0.50, 0.24), 0.524	-0.13 (-0.51, 0.24), 0.535	0.06 (-0.30, 0.41), 0.993	-0.16 (-0.60, 0.28), 0.611
Not Schedule 1	-0.01 (-0.05, 0.04)	-0.01 (-0.05, 0.03)	0.06 (0.01, 0.11)	-0.04 (-0.10, 0.02)
Schedule 1	-0.31 (-0.71, 0.09), 0.140	-0.35 (-0.75, 0.06), 0.105	-0.19 (-0.58, 0.20), 0.208	-0.37 (-0.86, 0.13), 0.205

Supplementary Table 6 Mean effect of extent of protected areas on population metrics of species of conservation concern (BoCC) or which are legally protected (Annex/Schedule 1), adjusted for the mean population size of species within the group. Asterisks indicate the significance of the difference in response to PA between species of conservation concern (red/amber-listed) and those not (green-listed) or for those designated under Annex 1 ('Birds' Directive) and Schedule 1 (UK Wildlife & Countryside Act) and those not so designated, after taking differences in population size into account (Exact P values after brackets and significant results, P < 0.05, are shown in bold.

	All PA	SSSI	SPA	SAC
**Occurrence**				
BoCC Green	-0.252 (-0.35, -0.16)	-0.274 (-0.38, -0.17)	-0.438 (-0.54, -0.34)	-0.271 (-0.36, -0.18)
BoCC Amber	0.467 (0.33, 0.61), 0.065	0.535 (0.38, 0.68), 0.057	**0.477 (0.34, 0.62), 0.002**	0.234 (0.11, 0.36), 0.23
BoCC Red	0.744 (0.49, 1), 0.155	0.858 (0.59, 1.13), 0.076	0.575 (0.33, 0.82), 0.078	0.495 (0.25, 0.73), 0.185
Not Annex 1	-1.44 (-1.78, -1.1)	-1.671 (-2.07, -1.27)	-1.651 (-2.01, -1.29)	-1.145 (-1.47, -0.82)
Annex 1	1.987 (1.54, 2.44), 0.861	2.306 (1.8, 2.81), 0.913	2.09 (1.63, 2.55), 0.405	1.448 (1.03, 1.86), 0.61
Not Schedule 1	-2.036 (-2.51, -1.56)	-2.272 (-2.79, -1.75)	-2.301 (-2.79, -1.81)	-1.571 (-2.01, -1.14)
Schedule 1	2.488 (1.93, 3.04), 0.472	2.762 (2.15, 3.37), 0.378	2.538 (1.98, 3.1), 0.537	1.74 (1.23, 2.25), 0.621
**Colonisation**				
BoCC Green	-0.577 (-0.67, -0.48)	-0.597 (-0.7, -0.5)	-0.627 (-0.73, -0.53)	-0.521 (-0.61, -0.44)
BoCC Amber	**0.39 (0.25, 0.53), 0.007**	**0.437 (0.29, 0.58), 0.003**	**0.39 (0.25, 0.53), < 0.001**	**0.12 (0, 0.24), 0.04**

BoCC Red	**0.629 (0.38, 0.88), 0.038**	**0.726 (0.47, 0.99), 0.01**	**0.504 (0.25, 0.76), 0.038**	0.314 (0.09, 0.54), 0.068
Not Annex 1	-2.05 (-2.42, -1.68)	-2.134 (-2.52, -1.75)	-2.011 (-2.4, -1.62)	-1.463 (-1.78, -1.15)
Annex 1	2.311 (1.84, 2.78), 0.611	2.455 (1.96, 2.95), 0.584	2.242 (1.76, 2.72), 0.282	1.392 (0.99, 1.8), 0.565
Not Schedule 1	-2.692 (-3.19, -2.19)	-2.811 (-3.33, -2.29)	-2.59 (-3.09, -2.09)	-1.91 (-2.34, -1.48)
Schedule 1	2.865 (2.3, 3.43), 0.936	3.036 (2.45, 3.62), 0.951	2.632 (2.08, 3.19), 0.837	1.767 (1.28, 2.25), 0.999
**Persistence**				
BoCC Green	-0.121 (-0.25, 0)	-0.104 (-0.23, 0.03)	-0.128 (-0.25, -0.01)	-0.095 (-0.21, 0.02)
BoCC Amber	0.509 (0.26, 0.76), 0.79	0.567 (0.3, 0.83), 0.816	0.602 (0.37, 0.84), 0.122	0.486 (0.26, 0.71), 0.542
BoCC Red	0.292 (-0.02, 0.6), 0.188	0.345 (0.02, 0.67), 0.156	0.16 (-0.14, 0.46), 0.432	0.346 (0.05, 0.64), 0.055
Not Annex 1	-0.796 (-1.09, -0.5)	-0.82 (-1.13, -0.51)	-0.698 (-0.98, -0.41)	-0.579 (-0.85, -0.31)
Annex 1	1.308 (0.56, 2.06), 0.617	1.353 (0.56, 2.14), 0.741	1.329 (0.67, 1.98), 0.362	1.216 (0.59, 1.84), 0.278
Not Schedule 1	-1.149 (-1.56, -0.74)	-1.192 (-1.62, -0.77)	-1.056 (-1.45, -0.66)	-0.886 (-1.26, -0.51)
Schedule 1	1.277 (-0.03, 2.58), 0.743	1.316 (-0.06, 2.69), 0.674	0.945 (-0.26, 2.15), 0.426	0.928 (-0.24, 2.1), 0.616
**Abundance**				
BoCC Green	-0.17 (-0.25, -0.09)	-0.17 (-0.25, -0.08)	-0.24 (-0.32, -0.16)	-0.21 (-0.29, -0.14)

BoCC Amber	**0.20 (0.02, 0.38), 0.029**	0.21 (0.03, 0.39), 0.027*	0.22 (0.05, 0.38), 0.481	0.17 (0.01, 0.32), 0.136
BoCC Red	0.58 (0.30, 0.85), 0.670	0.58 (0.30, 0.86), 0.753	0.52 (0.26, 0.78), 0.286	0.51 (0.26, 0.75), 0.328
Not Annex 1	-1.29 (-1.61, -0.97)	-1.31 (-1.64, -0.99)	-1.25 (-1.55, -0.95)	-1.16 (-1.44, -0.88)
Annex 1	1.64 (0.94, 2.33), 0.879	1.68 (0.97, 2.40), 0.889	1.59 (0.99, 2.18), 0.791	1.56 (1.01, 2.11), 0.539
Not Schedule 1	-1.52 (-1.90, -1.15)	-1.55 (-1.93, -1.17)	-1.48 (-1.84, -1.13)	-1.38 (-1.71, -1.04)
Schedule 1	1.67 (0.91, 2.44), 0.472	1.71 (0.93,2.49), 0.453	1.61 (0.96, 2.27), 0.602	1.58 (0.96, 2.21), 0.847
**Trend**				
BoCC Green	-0.03 (-0.08, 0.02)	-0.03 (-0.09, 0.02)	0.05 (-0.01, 0.11)	-0.07 (-0.15, 0.00)
BoCC Amber	-0.01 (-0.12, 0.10), 0.436	-0.01 (-0.12, 0.10), 0.404	0.07 (-0.05, 0.19), 0.681	-0.06 (-0.21, 0.09), 0.44
BoCC Red	0.08 (-0.09, 0.25), 0.082	0.07 (-0.11, 0.24), 0.111	0.10 (-0.09, 0.29), 0.534	0.06 (-0.18, 0.29), 0.079.
Not Annex 1	-0.00 (-0.20, 0.20)	0.00 (-0.20, 0.21)	0.05 (-0.18, 0.27)	0.02 (-0.26, 0.30)
Annex 1	-0.14 (-0.55, 0.27), 0.567	-0.14 (-0.56, 0.28), 0.589	0.07 (-0.35, 0.48), 0.968	-0.22 (-0.74, 0.29), 0.756
Not Schedule 1	-0.04 (-0.27, 0.20)	-0.04 (-0.28, 0.20)	-0.02 (-0.28, 0.24)	-0.02 (-0.35, 0.31)
Schedule 1	-0.29 (-0.73, 0.16), 0.144	-0.32 (-0.77, 0.13), 0.109	-0.12 (-0.57, 0.33), 0.164	-0.39 (-0.96, 0.18), 0.265

Supplementary Table 7 Percentage of bird species influenced by extent of protected area, dividing species into those with significant and non-significant (in parentheses) positive and negative correlations between population measures and area of designation based on the matched sample analysis. The χ^2 and P values relate to a two-sided test of proportion, testing the difference in proportion of species with significantly positive or negative correlations with area of designation. Significant results (P < 0.05) are shown in bold.

	**All Protected Areas**					**SSSI**					**SPA**					**SAC**				
	**+**	**(+)**	**(-)**	**-**	**χ^2, P**	**+**	**(+)**	**(-)**	**-**	**χ^2, P**	**+**	**(+)**	**(-)**	**-**	**χ^2, P**	**+**	**(+)**	**(-)**	**-**	**χ^2, P**
**Occurrence** (n=179)	**69**	**19.5**	**10.3**	**1.1**	**112.2, < 0.001**	**70.2**	**20.8**	**8.4**	**0.6**	**120.07, < 0.001**	**50.9**	**21.1**	**16.6**	**11.4**	**42.42, < 0.001**	**44.9**	**39.3**	**11.8**	**3.9**	**59.59, < 0.001**
**Colonisation** (n=165)	**38.7**	**31.2**	**24.3**	**5.8**	**40.73, < 0.001**	**38.1**	**33**	**23.3**	**5.7**	**40.73, < 0.001**	**29**	**35.8**	**21**	**14.2**	**8.22, 0.004**	**18**	**37.1**	**37.1**	**7.9**	**6.28, 0.012**
**Persistence** (n=130)	**24.6**	**45.9**	**22.1**	**7.4**	**10.26, 0.001**	**26**	**43.9**	**22**	**8.1**	**10.5, 0.001**	**18**	**41**	**33.6**	**7.4**	**4.65, 0.031**	**18.3**	**40.8**	**37.5**	**3.3**	**11.12, 0.001**
**Abundance** (n=133)	**47.4**	**19.6**	**7.2**	**25.8**	**5.63, 0.018**	**51**	**15.6**	**6.2**	**27.1**	**6.45, 0.011**	30.1	19.2	19.2	31.5	0, 1	36.8	22.4	11.8	28.9	0.5, 0.48
**Abundance Trend** (n=133)	11.3	39.2	33	16.5	0.59, 0.441	12.5	38.5	33.3	15.6	0.15, 0.7	23.3	34.2	31.5	11	2.56, 0.11	**11.8**	**27.6**	**31.6**	**28.9**	**4.65, 0.031**
**Productivity** (n=22)	13.6	27.3	18.2	40.9	2.08, 0.149	13.6	27.3	13.6	45.5	2.77, 0.096	11.8	29.4	35.3	23.5	0.17, 0.683	**6.2**	**12.5**	**31.2**	**50**	**4, 0.046**
**Productivity Trend** (n=22)	13.6	22.7	31.8	31.8	0.9, 0.343	9.1	27.3	31.8	31.8	1.78, 0.182	11.8	11.8	35.3	41.2	1.78, 0.182	6.2	31.2	31.2	31.2	1.5, 0.221

Supplementary Table 8 Mean association between demographic characteristics of individual species and protected area extent within the survey square (± standard error) from the statistically matched analysis, and (final column) the results of the two-sided t-test comparing the mean of SPA with SAC. P values are shown after each statistic and significant results (P < 0.05) are shown in bold.

	N Spp	All PA	SSSI	SPA	SAC	SPA vs SAC
**Occurrence**	178	**0.39 +/- 0.04, < 0.001**	**0.41 +/- 0.04, < 0.001**	**0.37 +/- 0.04, < 0.001**	**0.25 +/- 0.03, < 0.001**	**t = 4.2, >0.001**
**Colonisations**	178	**0.26 +/- 0.04, < 0.001**	**0.29 +/- 0.05, < 0.001**	**0.25 +/- 0.05, < 0.001**	**0.12 +/- 0.04, 0.001**	**t = 2.8, 0.005**
**Persistence**	122	**0.14 +/- 0.03, < 0.001**	**0.14 +/- 0.03, < 0.001**	**0.12 +/- 0.04, 0.003**	**0.1 +/- 0.05, 0.038**	t = 0.49, 0.622
**Abundance**	133	**0.15 +/- 0.03, < 0.001**	**0.14 +/- 0.03, < 0.001**	0.07 +/- 0.04, 0.083	**0.06 +/- 0.02, 0.024**	t = 0.03, 0.977
**Abundance trend**	133	0 +/- 0.02, 0.89	0 +/- 0.02, 0.801	0.03 +/- 0.03, 0.24	-0.05 +/- 0.03, 0.054	**t = 3.4, 0.001**
**Productivity**	22	**-0.11 +/- 0.05, 0.038**	**-0.11 +/- 0.05, 0.031**	-0.07 +/- 0.07, 0.29	**-0.27 +/- 0.11, 0.031**	t = 1.5, 0.153
**Productivity trend**	22	-0.04 +/- 0.04, 0.268	-0.04 +/- 0.04, 0.318	-0.08 +/- 0.08, 0.379	-0.11 +/- 0.06, 0.097	t = 0.22, 0.832

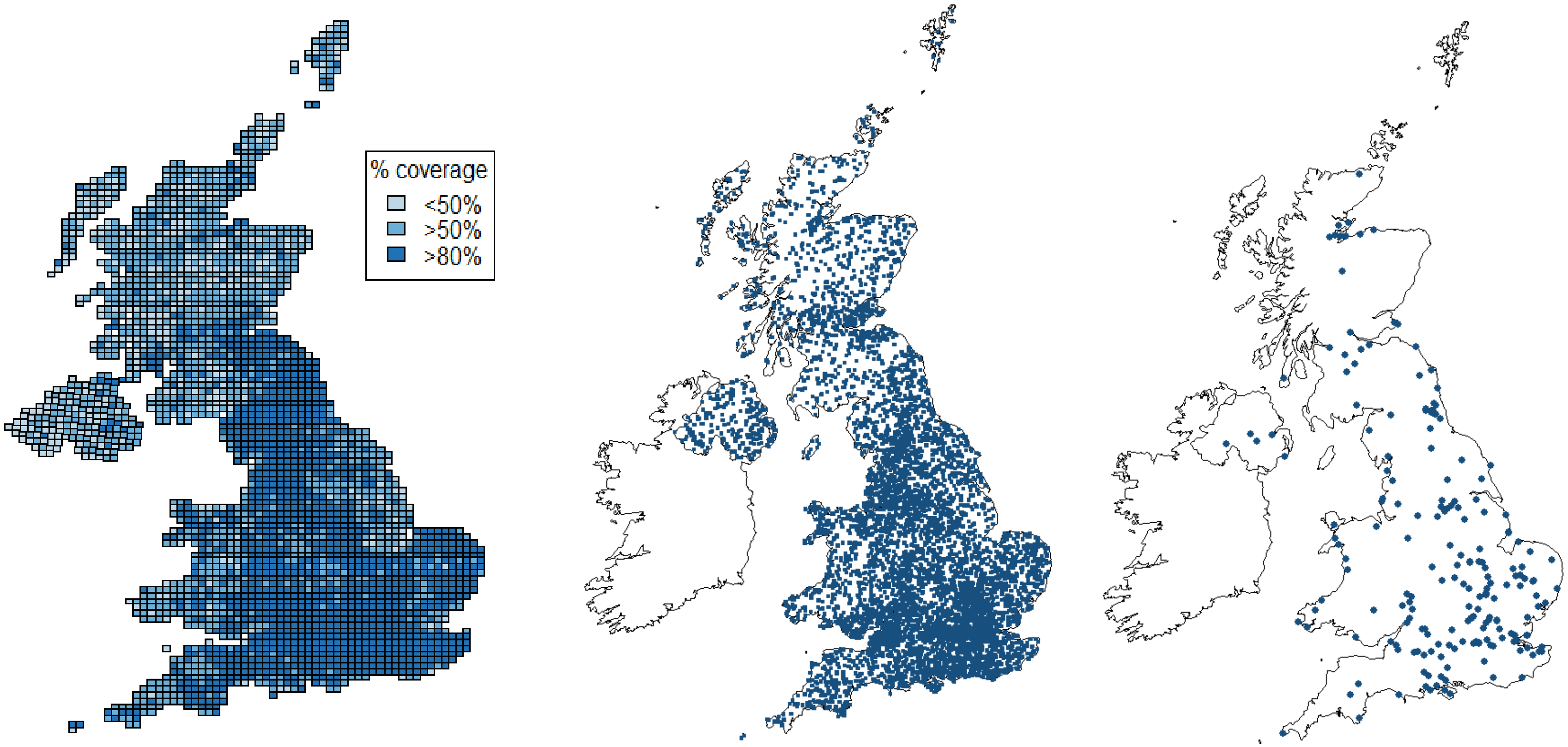

Supplementary Figure 1 Survey coverage. Percent of available 2km tetrads within each 10km square surveyed in either Atlas period (1988-91 or 2007-11, left), BBS survey squares (middle) and CES sites (right) contributing to the data. Contains Ordnance Survey data © Crown Copyright and database right 2022.

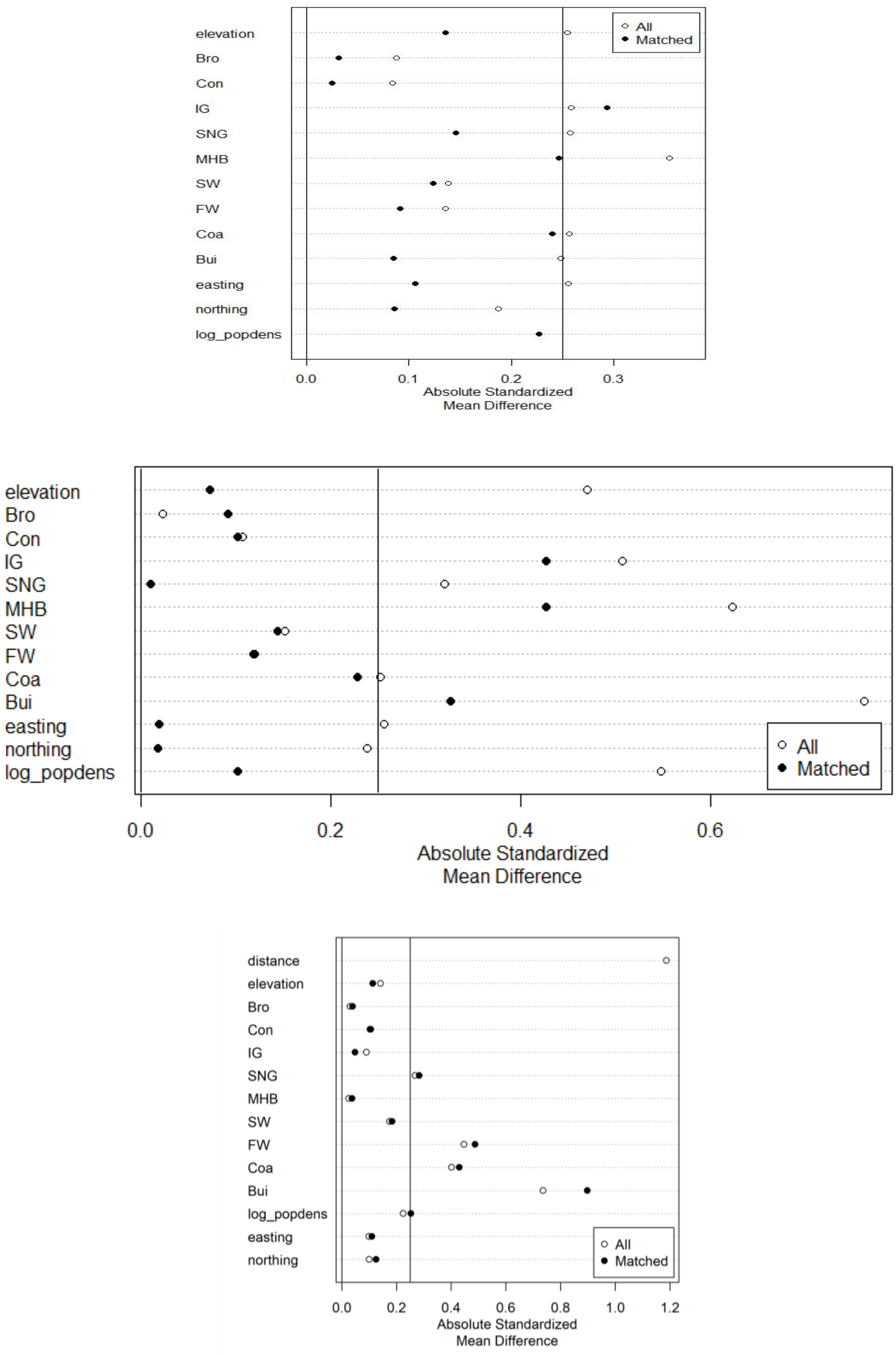

Supplementary Figure 2 Matching balance. The balance of samples along each variable axis before (open dots) and after (filled dots) matching for all PA in the Atlas (top), BBS (middle) and CES (bottom) datasets. Matching generally resulted in standardized mean differences below 0.25 (vertical line), which we considered well matched (Ho et al. 2007), however, because some imbalances were retained after matching we used covariate adjustments in the analysis of the

matched sample In some cases, the balance of samples was the same before and after matching so only one dot is visible.
