# Supplementary material for: Rare and declining bird species benefit most from designating protected areas for conservation in the UK
Source: Nat Ecol Evol. 2022 Dec 5;7(1):92–101. doi: 10.1038/s41559-022-01927-4 (PMC9834046; doi:10.1038/s41559-022-01927-4)
Supplement: Supplementary file 2 — Reporting Summary [file 41559_2022_1927_MOESM2_ESM.pdf]

nature portfolio

Corresponding author(s): Dr Robert A. Robinson and Dr Ailidh E. Barnes

Last updated by author(s): Jun 30, 2022

Reporting Summary

Nature Portfolio wishes to improve the reproducibility of the work that we publish. This form provides structure for consistency and transparency in reporting. For further information on Nature Portfolio policies, see our Editorial Policies and the Editorial Policy Checklist.

Statistics

For all statistical analyses, confirm that the following items are present in the figure legend, table legend, main text, or Methods section.

n/a	Confirmed	
☐	☒	The exact sample size (*n*) for each experimental group/condition, given as a discrete number and unit of measurement
☐	☒	A statement on whether measurements were taken from distinct samples or whether the same sample was measured repeatedly
☐	☒	The statistical test(s) used AND whether they are one- or two-sided<br>*Only common tests should be described solely by name; describe more complex techniques in the Methods section.*
☐	☒	A description of all covariates tested
☐	☒	A description of any assumptions or corrections, such as tests of normality and adjustment for multiple comparisons
☐	☒	A full description of the statistical parameters including central tendency (e.g. means) or other basic estimates (e.g. regression coefficient) AND variation (e.g. standard deviation) or associated estimates of uncertainty (e.g. confidence intervals)
☐	☒	For null hypothesis testing, the test statistic (e.g. F, t, r) with confidence intervals, effect sizes, degrees of freedom and P value noted<br>*Give P values as exact values whenever suitable.*
☐	☒	For Bayesian analysis, information on the choice of priors and Markov chain Monte Carlo settings
☐	☒	For hierarchical and complex designs, identification of the appropriate level for tests and full reporting of outcomes
☐	☒	Estimates of effect sizes (e.g. Cohen's *d*, Pearson's *r*), indicating how they were calculated

Our web collection on statistics for biologists contains articles on many of the points above.

Software and code

Policy information about availability of computer code

Data collection	No software was used in data collection, only data storage (Oracle) as the data were collected by citizen science volunteers.
Data analysis	All data was analysed in R using open sourced packages and functions and can be accessed on request via GitHub, https://github.com/BritishTrustForOrnithology/BirdsOnProtectedAreas.

For manuscripts utilizing custom algorithms or software that are central to the research but not yet described in published literature, software must be made available to editors and reviewers. We strongly encourage code deposition in a community repository (e.g. GitHub). See the Nature Portfolio guidelines for submitting code & software for further information.

Data

Policy information about availability of data

All manuscripts must include a data availability statement. This statement should provide the following information, where applicable:

- Accession codes, unique identifiers, or web links for publicly available datasets
- A description of any restrictions on data availability
- For clinical datasets or third party data, please ensure that the statement adheres to our policy

The datasets generated during and/or analysed during the current study are available in the Suppl File 1 (in figshare) repository, https://doi.org/10.6084/m9.figshare.20200895. The raw data is available on request from BTO.

Human research participants

Policy information about studies involving human research participants and Sex and Gender in Research.

Reporting on sex and gender	This data was not collected
Population characteristics	N/A
Recruitment	N/A
Ethics oversight	N/A

Note that full information on the approval of the study protocol must also be provided in the manuscript.

Field-specific reporting

Please select the one below that is the best fit for your research. If you are not sure, read the appropriate sections before making your selection.

☐ Life sciences ☐ Behavioural & social sciences ☒ Ecological, evolutionary & environmental sciences

For a reference copy of the document with all sections, see nature.com/documents/nr-reporting-summary-flat.pdf

Ecological, evolutionary & environmental sciences study design

All studies must disclose on these points even when the disclosure is negative.

Study description	Using volunteer collected survey data (see below) to assess whether measures of bird species were influenced by the proportion of protected area and what type of species (using traits) were mainly affected.
Research sample	Bird Atlas surveys of the UK avifauna undertaken in 1988-91 (Gibbons et al. 1993) and 2007-11 (Balmer et al. 2013), a total of 61,843 2-km squares.<br>Annual BTO/JNCC/RSPB Breeding Bird Survey data for the period 1994-2019 (Freeman et al. 2007) for a total of 6718 sites.<br>Constant effort mark-recapture program of birds (CES, Robinson et al. 2009) for the years 1990 (when 97 sites operated) through to 2019 (114 sites), with a total of 490 sites.
Sampling strategy	Sample sizes were chosen based on the relevant datasets, e.g. colonization from the Atlas dataset had to have the birds present in the second Atlas but not the first, and the removal of non-native species and seabirds (see below), and whether the species models converged during analysis. We used two approaches for analysis: i) a modelling approach that used all relevant data and included covariates to account for potentially confounding variables between PA and ii) statistical matching that used the same confounding variables to match PA sites with non-PA sites most similar to them. During the matching process sample sizes were reduced for the BBS and CES dataset as a result of their being insufficient 'control' squares to match to the "treatment" squares that contained PA.
Data collection	Atlas: volunteer surveyors record all adult birds seen using the 2-km square, in two timed visits (minimum 1 hour) in the breeding season (early visit - April - May, late visit - June - July).<br>BBS: volunteer surveyors record all adult birds they see or hear on two, 1km line-transects traversing a 1km square on two visits in the breeding season (early visit - 1st April-15th May and late visit - 16th May-30th June)<br>CES: volunteers erect mist-nets in set positions for a set length of time on, usually, 12 visits through the breeding season to capture all birds (adults and juveniles) at the site.
Timing and spatial scale	As above.
Data exclusions	Non-native bird species and seabirds were removed from the analysis as they do not report on them for trends and we cannot accurately census seabirds from these surveys.
Reproducibility	N/A: not an experiment
Randomization	N/A: not an experiment
Blinding	N/A: not an experiment

Did the study involve field work? ☐ Yes ☒ No

Reporting for specific materials, systems and methods

We require information from authors about some types of materials, experimental systems and methods used in many studies. Here, indicate whether each material, system or method listed is relevant to your study. If you are not sure if a list item applies to your research, read the appropriate section before selecting a response.

Materials & experimental systems

n/a	Involved in the study
☒	☐ Antibodies
☒	☐ Eukaryotic cell lines
☒	☐ Palaeontology and archaeology
☐	☒ Animals and other organisms
☒	☐ Clinical data
☒	☐ Dual use research of concern

Methods

n/a	Involved in the study
☒	☐ ChIP-seq
☒	☐ Flow cytometry
☒	☐ MRI-based neuroimaging

Animals and other research organisms

Policy information about studies involving animals; ARRIVE guidelines recommended for reporting animal research, and Sex and Gender in Research

Laboratory animals	Study did not involve laboratory animals.
Wild animals	The presence of wild birds were observed and recorded or captured and ringed in field sites across the UK by skilled volunteers following https://www.bto.org/our-science/bto-approach-science/animal-research-ethics; all animal capturing was undertaken by ringers by licensed by BTO, on behalf of the relevant statutory country body
Reporting on sex	This information was not collected for observations (BBS and Atlas) but was collected in the CES data, but not used in this analysis as productivity was per adult (male or female).
Field-collected samples	The study did not involve samples collected from the field.
Ethics oversight	All applicable national guidelines for the care and use of animals were followed according to https://www.bto.org/our-science/bto-approach-science/good-scientific-practice

Note that full information on the approval of the study protocol must also be provided in the manuscript.
